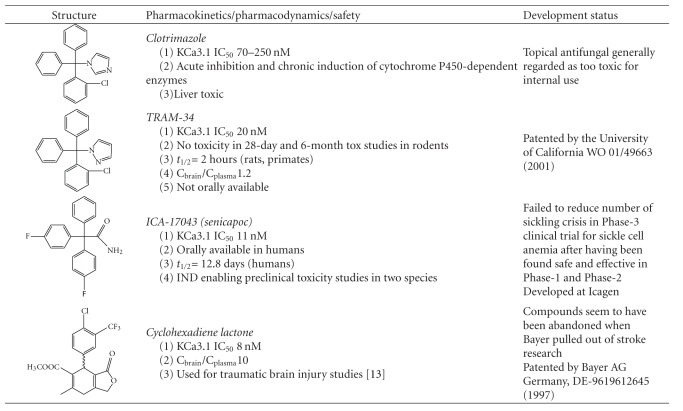# Microglial KCa3.1 Channels as a Potential Therapeutic Target for Alzheimer's Disease

**DOI:** 10.1155/2012/868972

**Published:** 2012-05-22

**Authors:** Izumi Maezawa, David Paul Jenkins, Benjamin E. Jin, Heike Wulff

**Affiliations:** ^1^Department of Pathology and Laboratory Medicine, University of California Davis, Davis, CA 95616, USA; ^2^MIND Institute, University of California Davis Medical Center, 2805 50th Street, Sacramento, CA 95817, USA; ^3^Department of Pharmacology, University of California Davis, Davis, CA 95616, USA

## Abstract

There exists an urgent need for new target discovery to treat Alzheimer's disease (AD); however, recent clinical trials based on anti-A**β** and anti-inflammatory strategies have yielded disappointing results. To expedite new drug discovery, we propose reposition targets which have been previously pursued by both industry and academia for indications other than AD. One such target is the calcium-activated potassium channel KCa3.1 (KCNN4), which in the brain is primarily expressed in microglia and is significantly upregulated when microglia are activated. We here review the existing evidence supporting that KCa3.1 inhibition could block microglial neurotoxicity without affecting their neuroprotective phagocytosis activity and without being broadly immunosuppressive. The anti-inflammatory and neuroprotective effects of KCa3.1 blockade would be suitable for treating AD as well as cerebrovascular and traumatic brain injuries, two well-known risk factors contributing to the dementia in AD patients presenting with mixed pathologies. Importantly, the pharmacokinetics and pharmacodynamics of several KCa3.1 blockers are well known, and a KCa3.1 blocker has been proven safe in clinical trials. It is therefore promising to reposition old or new KCa3.1 blockers for AD preclinical and clinical trials.

## 1. Repositioning an “Old,” Non-AD Specific Target for AD Therapy

All currently FDA-approved drugs for Alzheimer's disease (AD), the three acetylcholinesterase inhibitors Aricept, Razadyne, and Exelon, and the *N*-methyl-D-aspartate receptor antagonist, Namenda, only treat the symptoms of AD and cannot hold its progression. There therefore exists an urgent need for new target discovery to treat AD. The main approaches for AD drug discovery tend to focus on AD-specific molecular targets, such as those involved in the generation and aggregation of amyloid-*β* protein (A*β*). Several such targets have been investigated and have driven developments of therapeutic reagents showing impressive preclinical efficacy. However, very few of these developments have resulted in target validation in humans or successful translation to disease-modifying therapies [1].

These setbacks could still be overcome [2], but, in our view, should prompt pursuit of alternative approaches to target molecules that are not AD specific, which could provide additional chance of success. There are two major approaches under this category: either one could devise a broadly neuroprotective compound that is useful for many CNS indications, or one could “reposition” an existing target which has been previously pursued by both industry and academia for other indications and therefore is better understood. The former approach makes sense because it is increasingly obvious that AD is caused by multiple converging insults related but not specific to AD rather than by a single cascade pathway [3, 4]. This, in our view, is perhaps one of the reasons for failures of clinical trials targeting AD-specific pathways. We need to recall that the overwhelmingly major risk factor for AD is aging, and aging is certainly a multifactorial process. In addition, in reality, “pure AD” is relatively uncommon; most demented individuals show multiple pathologies including Lewy body pathology, TDP-43 pathology, and cerebrovascular diseases (CVDs), in addition to the traditional AD-type amyloid plaques and neurofibrillary tangles [5, 6]. In particular, the combination of CVD and AD commonly called “mixed dementia,” accounts for most dementia cases in community-dwelling older persons [7]. Approximately 60 to 90% of individuals with AD also have vascular brain pathologies [8]. Drugs specifically targeting pure AD pathologies may not address these common comorbidities, while broadly neuroprotective compounds could perhaps better address the downstream common pathways leading to synaptic and neuronal dysfunction. As an example, recently the Schubert group developed a drug screening procedure that is based upon old age-associated pathologies without requiring preselected molecular targets. The panel of screening assays was able to identify compounds that protect neurons from loss of trophic support, oxidative stress, aberrant energy metabolism, and amyloid toxicity. They subsequently identified a lead compound that showed promising effects in enhancing the memory performance of a transgenic AD mouse model [9]. Because the targets of this compound, although unknown, are not restricted to the AD-related amyloid toxicity pathway, this compound also facilitated memory in normal rodents. Viewed as a “memory enhancer,” this compound is predicted to be useful in other CNS indications affecting memory.

Our own approach, being reviewed here, is to reposition a target, KCa3.1, which has been pursued for both non-CNS and CNS indications for years, for AD therapy. We have to keep in mind that on average it currently takes at least 15 years and $1.5 billion to bring a drug for a major indication like AD to market. As an example, for more than a decade, remarkable efforts and resources have been devoted to anti-A*β* strategies based on the widely accepted amyloid cascade hypothesis [10]. However, results from several clinical trials are disappointing, for a multitude of reasons. In essence, Golde et al. pointed out that none of the putative anti-A*β* agents that have failed in pivotal phase 3 trials were optimal or even optimized agents within their class of anti-A*β* therapeutics [2]. They were hampered by low potency, poor brain penetration, and significant mechanism-based toxicity (such as from the nonselective action of *γ*-secretase inhibitors to block physiological functions), illustrating the practical difficulties of translating a brand new target to a new clinically useful drug. In addition, toxicity or poor tolerance during clinical trials is a common reason leading to failure of a new compound. It is therefore advantageous to reposition known targets, for which a wealth of pharmacological knowledge has been accumulated, and safety has been demonstrated in clinical trials. For these old targets, there typically exist useful pharmacological tool compounds that can be quickly resynthesized, evaluated in animal models to obtain proof-of-concept, and then optimized for specific properties such as brain penetration. This approach should expedite new drug development which is currently urgently needed for AD.

## 2. Three Criteria for Developing a “Pathway-Selective” Inhibitor of Microglial Activation for Anti-Inflammatory Therapy

Neuroinflammation and associated neuronal dysfunction mediated by activated microglia play an important role in the pathogenesis of AD [14]. Microglia, the resident macrophages and major mediator of neuroinflammation in the brain, can be activated by a variety of pathologic stimuli including the amyloid aggregates formed by amyloid-*β* protein (A*β*) [15–17]. Although microglia were initially noted to be abundantly present around amyloid plaques [18] and thought to be involved in plaque formation, recent positron emission tomography studies of patients with mild cognitive impairment (MCI) concluded that microglia activation occurs even before plaque and tangle formation [19] and is correlated with early cognitive deficits [20]. Although the exact stimuli that induce pathologic activation of microglia await further study, our recent results suggest that soluble A*β* oligomers (A*β*Os), the small and early-stage amyloid aggregates could be a potent stimulus [17]. A reasonable assumption is that multiple stimuli converge to cause microglial dysfunction and aberrant activation, thus aggravating microglia-mediated neurotoxicity and reducing their neuroprotective capacity. Indeed, a variety of life events, such as trauma, infection, stroke, metabolic disorders, and network hyperexcitability (epileptic seizures), have been implicated in contributing to the development of AD. Notably, all these conditions invariably activate microglia. Activated microglia release cytotoxic substances and proinflammatory cytokines to cause neuronal damage and age-associated microglial dysfunction [17, 21–23]. Above all, aging is perhaps the major risk and a prerequisite for “pathologic activation” that prevents microglia from performing the intended neuroprotective and repair functions [24, 25].

Irrespective of the events causing neuroinflammation in AD, curbing the harmful proinflammatory response of microglia activation is a reasonable approach toward prevention or therapy of AD. However, despite abundant preclinical evidence of their benefit, various anti-inflammatory approaches have not proven successful in clinical trials for a multitude of reasons. The most widely tested anti-inflammatory agents are the nonsteroidal anti-inflammatory drugs (NSAIDs). NSAIDs show multiple beneficial effects on preclinical cell culture and animal models of AD, although the exact molecular targets mediating these effects are not known. Unfortunately, results from several clinical trials are disappointing [26], partly due to inadequate CNS drug penetration of existing NSAIDs, suboptimal doses, unknown molecular targets (therefore unknown pharmacodynamics), and toxicities. For example, a recent large-scale AD prevention trial with NSAIDs, including naproxen and celecoxib, was stopped early because of drug safety concerns. Despite this, these setbacks should prompt investigations to develop novel anti-inflammatory agents with known specific targets, satisfactory CNS penetrance, and low toxicities.

We also have to consider that microglial activation can be neuroprotective through the release of neurotrophic factors and by phagocytosing A*β* and debris from degenerated neurons [14, 23]. Any anti-inflammatory therapies for AD should take these dichotomous microglial functions into consideration [23]. This constitutes our *criteria no.1* for microglia-targeted therapy in AD: the therapy should maintain microglial ability to migrate and clear A*β*, while inhibiting their release of neurotoxic mediators. Recent evidence suggests that this could be achieved by controlling the activities of specific pathways that can modulate certain aspects of microglia activation. Proposed approaches include modulation of the peroxisome proliferator-activated receptor-*γ* (PPAR-*γ*) [14] and the E prostanoid receptor subtype 2 [27]. Evidence obtained from our laboratory and from other groups strongly suggests blockade of the calcium-activated K^+^ channel KCa3.1 as another promising approach that could curb inflammatory brain pathologies while preserving microglial migration and phagocytosis [13, 17, 28–30].

Two additional criteria for an anti-inflammatory drug for AD therapy are: *criteria no.2*, it should be relatively specific to microglia to avoid adverse neuronal effects and *criteria no.3*, it should not be broadly immunosuppressive. We will review evidence supporting that KCa3.1 is a suitable therapeutic target for AD using the above three criteria.

## 3. KCa3.1 as a Microglia-Selective Target in CNS

**Figure 1:**
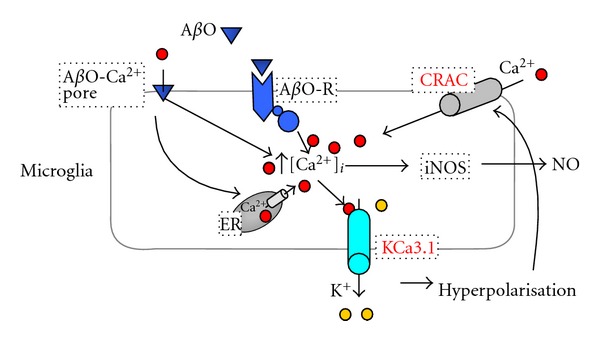
KCa3.1 regulates microglial activation by modulating Ca^2+^ influx. A*β*O initiates an increase of intracellular Ca^2+^ either directly by forming a Ca^2+^-permeable membrane pore (A*β*O-Ca^2+^ pore) [11, 12] or indirectly through interaction with a receptor (tentatively termed A*β*O-R). Intracellular Ca^2+^ activates KCa3.1 to induce K^+^ efflux. The resulting hyperpolarisation provides the driving force for Ca^2+^ entry through store-operated inward-rectifier calcium channels like CRAC, thus sustaining the Ca^2+^ signal necessary for selective Ca^2+^ activated pathways. One example illustrated here is iNOS activation and nitric oxide (NO) production to cause microglia-mediated neurotoxicity.

K^+^ channels are encoded by a super-family of 78 genes [31] and are involved in diverse physiological and pathological processes [32]. K^+^ channels accordingly already serve as drug targets for cardiac arrhythmia, type-2 diabetes, and epilepsy and have been proposed as potential targets for various neurological diseases such as multiple sclerosis, Parkinson's disease, stroke, pain, schizophrenia, and migraine. Two K^+^ channels, the calcium-activated KCa3.1 (also known as IK1, SK4 or KCNN4) and the voltage-gated Kv1.3, play important roles in microglia activation by modulating Ca^2+^ signaling and membrane potential. Similar to T cells, where their roles have been studied in much more detail [33], K^+^ efflux through microglial KCa3.1 and Kv1.3 helps maintain a negative membrane potential for Ca^2+^ influx through the store-operated inward rectifier calcium channel CRAC (Ca^2+^ release activated Ca^2+^ channel) (Figure 1). However, the two K^+^ channels appear to be differentially expressed following immune cell activation and differentially modulate cytokine production and cellular proliferation in different T and B cell subsets. While Kv1.3 is primarily important in CCR7^−^ effector memory T cells and class-switched IgD^−^CD27^+^ B cells, CCR7^+^ T cells and IgD^+^ B cells rely on KCa3.1 for part of their calcium-signaling and activation events [34–37]. In microglia, KCa3.1-mediated control of Ca^2+^ entry has been shown to be involved in oxidative burst, nitric oxide production, and microglia-mediated neuronal killing, including that induced by A*β* oligomer (A*β*O) [17, 28, 38, 39].

Although no human diseases involving KCa3.1 mutations have been described so far, KCa3.1 constitutes a very attractive and (in some cases) relatively well-validated drug target for diseases or conditions ranging from sickle cell disease, restenosis, and atherosclerosis to asthma and traumatic brain injury (see [40] for a recent review). KCa3.1 channels are widely expressed throughout the body and primarily found in hematopoietic-derived cells including macrophages/microglia. A significant advantage of KCa3.1 channels as a therapeutic target for CNS indications is that expression seems to be restricted to hematopoietic-derived cells and peripheral tissues such as secretory epithelial, fibroblasts, and proliferating neointimal smooth muscle cells [40], but the channels have been found to be absent from excitable tissues such as neurons and cardiomyocytes [41–43]. KCa3.1 channels appear also not expressed in astrocytes. However, evidence shown in a recent article by Bouhy et al. suggests expression of KCa3.1 in reactive astrocytes in the spinal cord of a mouse spinal cord injury model, although only one anti-KCa3.1 antibody was used in immunohistochemistry [30]. We feel that this is clearly not the case for the forebrain, based on previously published gene expression data [41, 43]. Our own experiments using several polyclonal and monoclonal anti-KCa3.1 antibodies to stain sections from models of AD (unpublished results) and stroke [29] show that KCa3.1 is substantially upregulated in activated microglia, but not in astrocytes. We therefore feel that we can perhaps conclude that the major parenchymal cells in the cerebrum in which KCa3.1 channels play a significant role are microglia, and in some pathological conditions, invading macrophages. Therefore, it is reasonable to assume that a CNS-permeating KCa3.1 blocker would have relatively selective actions on microglia and would avoid adversely affecting neuronal functions.

## 4. KCa3.1 Blockers Are Neuroprotective

Recognizing the important role of KCa3.1 in regulating immune cell functions, Wulff et al. synthesized a specific KCa3.1 blocker called TRAM-34 using as a template the antimycotic clotrimazole, which is a potent but poorly tolerated KCa3.1 inhibitor [44]. TRAM-34 (IC_50 _20 nM) is currently the most widely used pharmacological tool compound for studying the pathophysiology of KCa3.1 because of its high selectivity over other K^+^ channels and its availability to academic researchers.  Table 1 shows the structures of TRAM-34 and several other KCa3.1 blockers developed by pharmaceutical companies and summarizes pharmacokinetic, safety, and development information. For more extensive reviews on KCa3.1 pharmacology, interested readers are referred to two review articles [40, 45].

 TRAM-34 has been tested in various animal models, including optic nerve transaction [28], middle cerebral artery occlusion [29], traumatic brain injury [13] and restenosis [46] in rats; traumatic spinal cord injury [30], atherosclerosis [47], and inflammatory bowel disease [37] in mice; and angioplasty in pigs [48]. In particular, the following *in vivo* observations taken together provide strong evidence that KCa3.1 inhibitors can curb brain inflammation and provide neuroprotection.

1. Our own group recently demonstrated that TRAM-34 inhibits A*β*O-induced microglia activation and microglia-mediated neuronal toxicity [17].
2. Our group further showed that TRAM-34 inhibits microglia activation and reduces infarct area and neurological deficit scores in a rat model of ischemic stroke even if treatment is commenced 12 h after reperfusion [29].
3. The Schlichter group showed that TRAM-34 reduces retinal ganglion cell degeneration after optic nerve transection in rats [28]. Interestingly, KCa3.1 blockade did not prevent microglia from aligning with damaged axons or from phagocytosing damaged neurons, but increased the number of surviving retinal ganglion cells presumably by reducing the production and/or secretion of neurotoxic molecules in the retina [28]. This could possibly be explained by the observation that the Ca^2+^ influx during phagocytosis appears to be mediated through reverse mode Na^+^/Ca^2+^ exchange [49] and not through KCa3.1-regulated CRAC channels, supporting the “pathway-selective” nature of KCa3.1 inhibition.
4. The David group showed that TRAM-34 reduces the secondary damage and improves locomotor function in a mouse model of spinal cord injury in a dose-dependent manner [30].
5. Scientists at Bayer demonstrated that two structurally different KCa3.1 inhibitors, a triarylmethane and a cyclohexadiene (Table 1), reduced infarct volume and brain edema following traumatic brain injury caused by acute subdural haematoma in rats [13].
6. Scientists at Schering resynthesized TRAM-34 and showed that it treats MOG-induced experimental autoimmune encephalomyelitis in mice by reducing the production of the inflammatory cytokines INF-*γ* and TNF-*α* in the brain and spinal cord [50].

## 5. Targeting KCa3.1 Could Ameliorate A***β***O-Induced Neuronal Damage

A*β*Os, the small soluble and diffusible aggregates of A*β* peptides, were initially considered transient or metastable intermediates in fibril formation [51]. However, some of them may not be obligate intermediates in the fibril formation pathway and can be stable [52, 53]. Importantly, recent* in vitro* and *in vivo *studies have revealed that the buildup of soluble A*β*O may be an early and central event in the pathogenesis of AD [54–57]. The strong and rapidly disruptive effect of A*β*O on synaptic plasticity and neuronal integrity is hypothesized to cause memory problems in AD and is generally attributed to their direct neuro- or synaptotoxicity [10]. However, one plausible but less studied possibility is that A*β*O activates microglia and causes indirect, microglia-mediated neuro- and synaptotoxicity. Recently we found that A*β*O, either assembled *in vitro* from synthetic A*β*1-42 peptide or isolated from AD brains, is a highly potent activator of microglia [17]. Although the mechanism mediating A*β*O-induced microglia activation and the exact pattern of activation are still under investigation, a particularly interesting observation is that this mode of microglia activation and related neurotoxicity are dependent on microglial KCa3.1. We found that TRAM-34 blocked A*β*O-induced microglia proliferation, p38MAPK phosphorylation, NF*κ*B activation, and nitric oxide generation. We further showed that the neurotoxic effects of low concentrations of A*β*O (10–50 nM) applied to mixed microglia-neuron cultures or organotypic hippocampal slices were almost completely blocked by cotreatment with TRAM-34, another microglial activation inhibitor doxycycline, and inhibitors of iNOS. This set of results suggests that A*β*O, although generally considered a neurotoxin, may more potently cause indirect neuronal damage by activating microglia in AD. Consistent with this notion, a previous study showed that the inhibition of NMDA receptor-dependent long-term potentiation by soluble A*β* can be prevented by minocycline, a microglia activation inhibitor in the same class as doxycycline, and iNOS inhibition to reduce nitric oxide production from microglia [58]. Taken together, these results suggest that KCa3.1 blockers could potentially also inhibit microglial neurotoxicity and thus preserve memory in AD.

## 6. Targeting KCa3.1 Could Also Effectively Address Cerebrovascular and Traumatic Comorbidities in AD

As discussed above, cerebrovascular insults and traumatic brain injuries are significant comorbidities in AD. In addition to clinically apparent strokes, carotid, vertebral, and intracranial vascular stenosis can cause chronic cerebral hypoperfusion, microinfarcts, and lacunar infarcts, contributing to dementia. These vascular and traumatic pathologies cannot possibly be addressed by AD-specific therapies, such as antiamyloid drugs or vaccines. The well-documented beneficial effects of KCa3.1 blockers in models of ischemic stroke [29], traumatic brain injury [13], and atherosclerosis [47], which primarily seem to be mediated through inhibition of detrimental microglia/macrophage function, considerably add to KCa3.1′s attractiveness as a novel target for treating the dominant group of AD patients presenting with both degenerative and vascular pathologies. KCa3.1 is further expressed in dedifferentiated, proliferative vascular smooth muscles cells, which, as Köhler et al. showed, switch from their normal KCa1.1 (BK) channel expression to KCa3.1 expression following balloon catheter injury. In keeping with a role of KCa3.1 in driving aberrant smooth muscle cell proliferation, TRAM-34 prevents vascular restenosis in a rat model [46]. These findings were more recently confirmed by a study in which coating of TRAM-34 onto balloon catheters significantly reduced restenosis in pigs, which very closely resemble humans with respect to postangioplasty restenosis [48]. A similar increase in KCa3.1 expression was found in coronary vessels from patients with coronary artery disease and in aortas from ApoE^−/−^ mice, suggesting that KCa3.1 is involved in atherogenesis. KCa3.1 blockade with TRAM-34 prevented atherosclerosis development in ApoE^−/−^ mice by reducing smooth muscle cell proliferation and macrophage infiltration into atherosclerotic plaques [47]. Furthermore, TRAM-34 administration reduced the inflammatory neurotoxicity and infarct areas in the wake of ischemic stroke, even when the first dose was applied at 12 hours after reperfusion. This was accompanied by a dose-dependent improvement in neurological deficit score, a reduction in the number of ED1^+^ activated microglia and an increase in NeuN^+^ surviving neurons [29].

## 7. KCa3.1 Blockers Are Mild Immunosuppressants and Are Relatively Safe

The promise of KCa3.1 as a therapeutic target for AD is further strengthened by the observations that KCa3.1 blockers are very mild immunosuppressants that do not reduce the ability of rodents to clear viral infections like flu [47]. In addition, genetic or pharmacological blockade of KCa3.1 seems relatively safe and well tolerated. Two independently generated KCa3.1^−/−^ mice were both viable, of normal appearance, produced normal litter sizes, did not show any gross abnormalities in any of their major organs, and exhibited rather mild phenotypes: impaired volume regulation in erythrocytes and lymphocytes [59], a reduced EDHF (endothelium derived hyperpolarizing factor) response together with a mild ~7 mmHg increase in blood pressure [60], and subtle erythrocyte macrocytosis and progressive splenomegaly [61]. A 28-day toxicity study with TRAM-34 in mice resulted in no observable changes in blood chemistry, hematology or necropsy of any of the major organs [47]. A subsequent 6-month toxicity study with TRAM-34 in rats also did not find any changes in the same parameters and also did not report any increases in susceptibility to viral or bacterial infections [29]. Senicapoc, a KCa3.1 blocker structurally similar to TRAM-34 (see Table 1), was safe and well tolerate, in a Phase-1 clinical trial in healthy volunteers [62] and was afterwards found to significantly reduce hemolysis and increase hemoglobin levels in a 12-week, multicenter, randomized double-blind Phase-2 study in sickle cell disease patients [63]. However, in a subsequent Phase-3 study, which was designed to compare the rate of acute vasoocclusive pain crisis occurring in sickle cell disease patients, Senicapoc failed to reduce this desired clinical endpoints despite again reducing hemolysis and increasing hemoglobin levels and not inducing any significant adverse events (see [40] for a more extensive discussion of the clinical experiences with KCa3.1 blockers).

## 8. Conclusion: Microglial KCa3.1 Is a Promising Target for AD

Concluding the above discussion, we here propose that microglial KCa3.1 is a promising therapeutic target for AD because KCa3.1 blockade comes close to fulfilling three criteria we set for anti-inflammatory therapy. Using the specific KCa3.1 inhibitor TRAM-34 as a pharmacological tool compound, proof-of-concept studies have shown that KCa3.1 inhibition can reduce A*β*O-induced microglial neurotoxicity and protect neurons in other non-A*β* neuronal injury models by reducing the production of neurotoxic proinflammatory mediators while preserving the neuroprotective functions of microglia, such as migration and phagocytosis. This “pathway-selectivity” is likely due to the ability of KCa3.1 to “fine-tune” the pattern of microglial activation by selectively regulating various Ca^2+^-activated signaling pathways. Due to its demonstrated effects on models of CVD and traumatic brain injuries, two well-known risk factors for AD, KCa3.1 inhibition could offer additional therapeutic benefits for mixed pathologies commonly seen in AD patients. KCa3.1 blockade by either pharmacological inhibition or genetic knockout only resulted in minimal immunosuppression. Importantly, a KCa3.1 blocker has been proven safe in clinical trials. Therefore, it is promising to either directly reposition existing KCa3.1 blockers for AD preclinical proof-of-concept studies and subsequent clinical trials and/or make efforts to optimize existing or newly-discovered compounds for oral availability and brain penetration in order to expedite drug development for AD.

## Figures and Tables

**Table 1:**